# Diabetic Muscle Infarction of the Tibialis Anterior and Extensor Hallucis Longus Muscles Mimicking the Malignant Soft-Tissue Tumor

**DOI:** 10.1155/2015/656307

**Published:** 2015-07-05

**Authors:** Yoshikuni Mimata, Kotaro Sato, Karen Tokunaga, Itsuko Tsukimura, Hiroshi Tada, Minoru Doita

**Affiliations:** Department of Orthopedic Surgery, School of Medicine, Iwate Medical University, Morioka 020-8505, Japan

## Abstract

One of the most common causes of skeletal muscle infarction is diabetic muscle infarction (DMI), a rare complication associated with poorly controlled diabetes. We report an atypical case of DMI localized in the tibialis anterior (TA) and extensor hallucis longus (EHL) muscles of an elderly individual. A 64-year-old man with type 2 diabetes mellitus presented with a 6-month history of a palpable mass in his lower left leg. Magnetic resonance imaging (MRI) revealed that the mass exhibited heterogeneous signals on T1- and T2-weighted images and slight heterogeneous enhancement within the muscles on fat suppressed T1-weighted images. Because histopathological analysis revealed mostly necrotic muscle tissues but no neoplastic cells, we resected the affected muscles. A typical symptom of DMI is severe abrupt-onset pain in the region of the affected muscles, but the patient did not complain of pain. Therefore, the diagnosis and treatment for DMI were delayed, and widespread irreversible muscle necrosis developed. MRI findings of DMI can be similar to that of a malignant soft-tissue tumor. So, it is necessary to consider the malignant soft-tissue tumor as one of the differential diagnoses of DMI.

## 1. Introduction

Skeletal muscle infarction is ischemic necrosis of striated muscles of the skeleton due to long-term poorly controlled diabetes mellitus, alcoholism, compartment syndrome after blunt trauma or fracture, and vascular diseases such as acute artery thrombosis or embolism and arteriosclerosis obliterans (ASO). The typical clinical symptom is severe abrupt-onset pain in the region of the affected muscle.

Herein, we describe a rare case of diabetic muscle infarction (DMI) localized in the region of the tibialis anterior (TA) and extensor hallucis longus (EHL) muscles. The patient's clinical symptom and magnetic resonance imaging (MRI) findings were atypical and interesting compared to those of previously reported patients with DMI. The patient was aware of a palpable mass in the lower left leg for 6 months but neglected it owing to a lack of pain. A palpable mass was suggestive of a malignant soft-tissue tumor; therefore, we needed to distinguish the lesion from a malignant soft-tissue tumor.

## 2. Case Report

**Figure 1:**
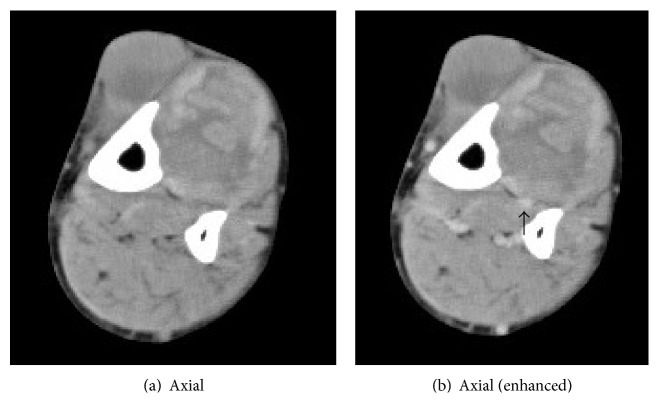
Computed tomography (CT) of left lower leg revealed a large mass (maximum diameter, 7.2 cm; length, 21.0 cm) with cystic lesions in the region of the TA and EHL muscles (a and b). The mass and cystic lesions were not contrasted but the anterior tibialis artery was clearly observed (arrow) by using enhanced CT (b).

**Figure 2:**
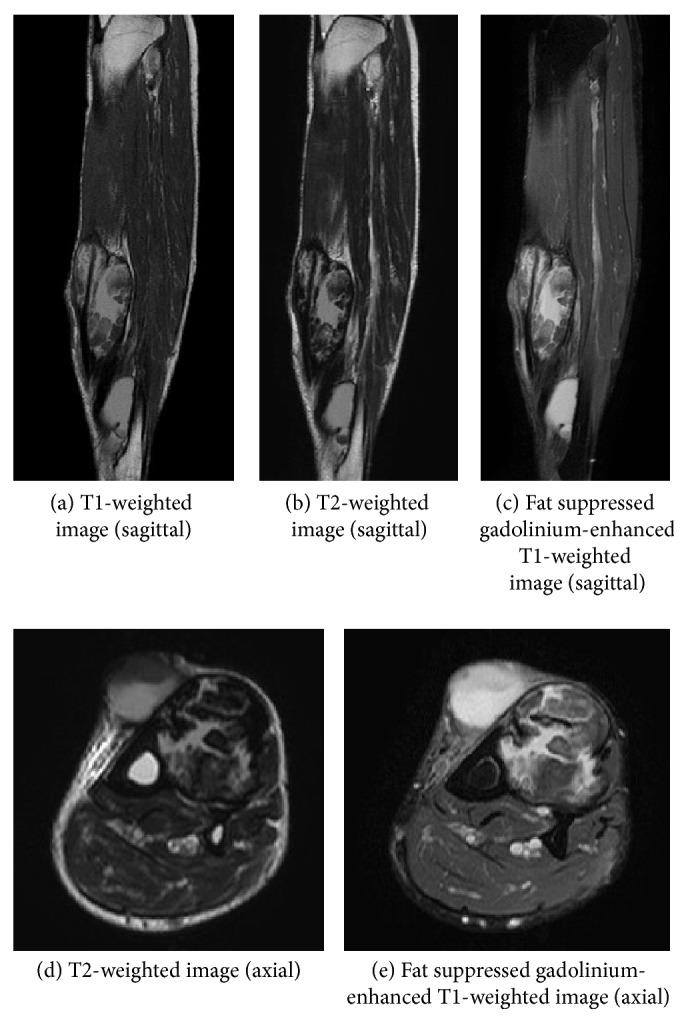
MRI revealed that the mass exhibited heterogeneous signals on T1- and T2-weighted images and the cystic lesions exhibited homogeneous signals on T1- and T2-weighted images (a, b, and d). Fat suppressed gadolinium-enhanced T1-weighted images revealed slight heterogeneous enhancement within the affected muscle with focal hypointense nonenhancing areas (c and e).

**Figure 3:**
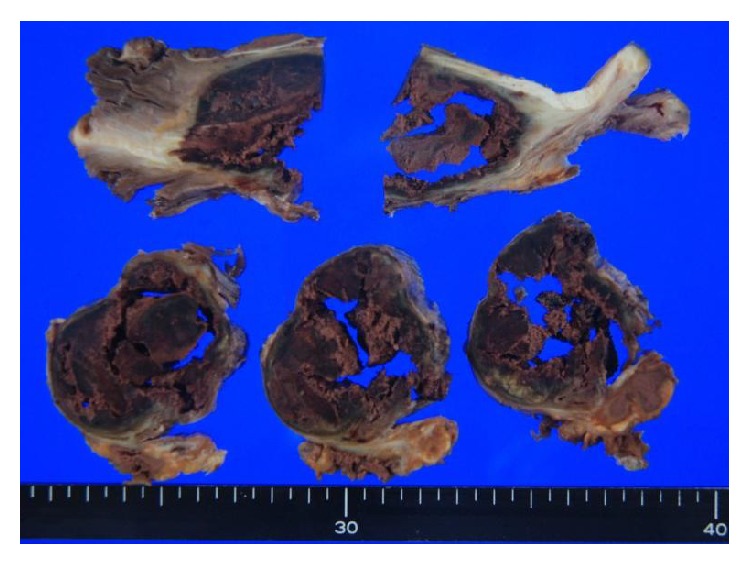
Macroscopic findings. Macroscopic findings revealed diffuse necrosis of tibialis anterior and extensor hallucis longus muscles.

**Figure 4:**
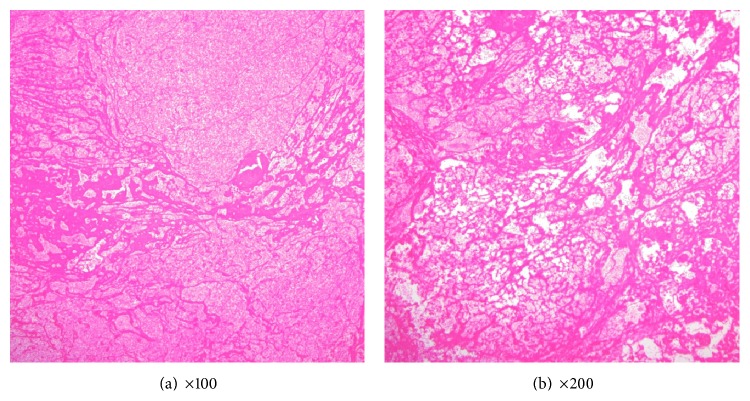
Histopathological findings (HE staining). Histopathological analysis revealed mostly necrotic muscle tissues, but neoplastic cells were not observed in the soft tissue resected from the lesion (a and b).

A 64-year-old man was referred to our hospital with the diagnosis of a soft-tissue tumor in the lower left leg. He had a 10-year history of type 2 diabetes mellitus, and he was consuming oral hypoglycemic agents. However neither nephropathy nor retinopathy was pointed out by clinical examination such as blood test, urine test, and funduscopy before, and he did not complain of sensory disturbance. He also had a history of reconstruction of an acute aortic dissection. He was aware of a palpable mass in his lower left leg but had neglected it for 6 months because he did not feel pain at the site of the lesion. The mass gradually expanded and developed large blisters. Physical examination revealed swelling without tenderness on the anterior aspect of the lower left leg. Blood tests revealed the following: C-reactive protein level, 0.05 mg/L; white blood cell count, 4,580/mm^3^; CK level, 164 IU/L; and casual blood glucose level, 298 mg/dL. Radiography revealed no bony abnormalities and calcification of soft tissue, but there was evidence of moderate soft-tissue swelling. Computed tomography (CT) revealed a large mass (maximum diameter, 7.2 cm; length, 21.0 cm) with cystic lesions in the region of the TA and EHL muscles (Figure 1). Neither the calcification nor ossification was detected inside and around the mass. The mass and cystic lesions were not contrasted but the anterior tibialis artery was clearly observed by using enhanced CT. MRI revealed that the mass exhibited heterogeneous signals on T1- and T2-weighted images and the cystic lesions exhibited homogeneous signals on T1- and T2-weighted images; perifascial, intramuscular, and/or subcutaneous edema was not seen (Figure 2). Fat suppressed gadolinium-enhanced T1-weighted images revealed slight heterogeneous enhancement within the affected muscle with focal hypointense nonenhancing areas. The ankle brachial index (ABI) was normal. To distinguish muscle necrosis from a malignant soft-tissue tumor, a needle biopsy (Tru-Cut biopsy needle, 14-gauge, CareFusion, CA, USA) was performed. However, we did not collect full volume of the muscle tissue; therefore, open surgical muscle biopsy was performed. Histopathological analysis revealed mostly necrotic muscle tissues, but neoplastic cells were not observed in the soft tissue resected from the lesion. Because the lesion was a remnant of a previous muscle infarction with several large blisters producing discharge, we resected the affected muscles (Figure 3). Excisional specimens revealed large areas of muscles with coagulative necrosis, fibrosis, and hemorrhage (Figure 4). Two weeks after surgery, the wound healed, and the patient was discharged. There is no evidence of any diseases including local recurrence at 9 months after surgery.

## 3. Discussion

The most common causes of skeletal muscle infarction are vascular diseases such as artery thrombosis or embolism and ASO. Another cause is a rare complication of diabetes mellitus. It is very important to distinguish these diseases correctly because methods of treatment for each disease are different.

DMI is a rare complication associated with long standing and poorly controlled diabetes mellitus; it was first reported by Angervall and Stener in 1965 [1]. A systematic review by Trujillo-Santos and colleagues in 2003 showed that DMI was more frequent in women (61.5%), with a mean age at presentation of 42.6 years and a mean duration of diabetes of 14.3 years [2]. Approximately 60% of the patients had had type 1 diabetes, and approximately 24% had type 2 diabetes [2]. However, a systematic review by Horton and colleagues in 2015 showed there was not type 1 diabetes or female predominance in patients with DMI [3]. The most frequently affected site of DMI was the thigh (71.2–83.7%), while the calf was the second most commonly affected site (15.3–19.3%) [2, 3]. Horton reported that the incidence rate of DMI in TA and EHL muscles was 2.9% and 0.7%, respectively, and so our reported case of DMI localized in TA and EHL muscles was very rare. Vascular complications of diabetes were reported in most patients, mostly nephropathy (71.1%), retinopathy (56.6%), and neuropathy (54.5%) [2]. Our patient had a 10-year history of poorly controlled type 2 diabetes mellitus, but neither nephropathy nor retinopathy was pointed out by clinical examination such as blood test, urine test, and funduscopy before and he did not complain of sensory disturbance.

The pathogenesis of DMI may involve atherosclerotic occlusion, hypoxia-reperfusion injury, vasculitis with thrombosis, arterial embolism of small vessels, and a hypercoagulable state [2–7]. However, it is not fully understood and is controversial. In diabetic microangiopathy, high blood glucose levels cause the endothelial cell lining of the blood vessels to absorb more glucose than normal, resulting in vascular fragility. In our case, enhanced CT revealed that the tibialis anterior artery was clearly visualized, and the ABI was normal. Therefore, we assume that atherosclerotic occlusion and arterial embolism of small vessels caused by microangiopathy led to the onset of DMI.

Typical symptoms of DMI can include local swelling, abrupt onset of pain in the affected muscle, and a palpable painful mass [2–7]. If the area of necrosis is wide, muscle weakness may occur in the area where the muscle tissue necrotizes. Patients with diabetes often have diabetic peripheral neuropathy, a complication in which the peripheral nervous system breaks down, and they lose sensation in their extremities. Therefore, DMI can become severe because patients with diabetes neglect their lesions owing to a lack of pain. Interestingly, our patient had no complaint of pain in his lower leg even though he was aware of a palpable mass for 6 months. According to the clinical course and physical findings, the lesion simulated a malignant soft-tissue tumor more closely than a muscle infarction.

Other differential diagnoses except soft-tissue tumor included muscle infection (cellulitis, abscess, and necrotizing fasciitis) and calcific myonecrosis which is characterized by a slowly progressive enlarging, painless soft-tissue mass with occasional tenderness in the lower limb. The most common area of calcific myonecrosis is the anterolateral part of the lower leg. Although this condition is not fully understood, it is assumed that these lesions most likely result from posttraumatic ischemia and cystic degeneration of the muscle. Radiographs typically show a fusiform mass with peripherally oriented plaque-like amorphous calcifications. In our patient, the calcification was not seen by radiographs and CT, and so, we ruled out this disease. In cellulitis, subcutaneous swelling is seen but with no muscle involvement [8]. Necrotizing fasciitis has MR findings including muscle swelling, edema, and inflammatory changes similar to DMI. However, compared to DMI, necrotizing fasciitis has less pronounced muscle involvement and more extensive fascial involvement [8]. Additionally, gas bubbles and fluid collection may be seen in the tissues [8].

To confirm the correct diagnosis, the clinical course, physical findings, CT and MRI features, and histopathological analysis are important. Muscle biopsy is the gold standard for diagnosis and exclusion of other diseases such as pyomyositis, necrotizing fasciitis, and soft-tissue tumors [8–10]. However, recent studies reported that surgical biopsy would either prolong the disease in the individual or acutely exacerbate this condition [2, 3, 9]. Chason and Kiers mentioned that MRI was the most sensitive modality, as it was very useful for diagnosis and could avoid the need for biopsy [9, 10]. As such, a needle biopsy may be reserved for cases in which the clinical presentation is atypical or the diagnosis is uncertain. In our patient, MRI revealed localization of the lesion only in the TA and EHL muscles, suggesting a previous muscle necrosis or a malignant soft-tissue tumor. To rule out a malignant soft-tissue tumor, we performed a muscle biopsy. Histopathological analysis showed that the necrotic and atrophic skeletal muscle had been infiltrated by necrotic neutrophils. On the basis of the above results, we diagnosed the lesion as a DMI. Because the lesion was a remnant of a previous muscle infarction with several large blisters producing discharge, we resected the affected muscles.

In summary, herein, we reported a rare case of DMI in the TA and EHL muscles. The typical symptom of DMI is severe abrupt-onset pain in the region of the affected muscle, but our patient did not complain of pain. The diagnosis of and treatment for DMI were delayed, and widespread irreversible muscle necrosis requiring resection developed. It is true that MRI is the most sensitive test for making the diagnosis of DMI; however, in some cases, MRI finding of DMI can be similar to that of the malignant soft-tissue tumor. So, it is necessary to consider the malignant soft-tissue tumor as one of the differential diagnoses of DMI.
